# Evaluation of infants aged 0–12 months admitted to the pediatric emergency department: the example of Batman Training And Research Hospital

**DOI:** 10.1590/1806-9282.20260588

**Published:** 2026-10-05

**Authors:** Semih Canpolat, Mehmet Kenan Nahya, Ertuğrul Dedeoğlu, Mesut Saka, Mehmet Emin Parlak, Aylin Aktı

**Affiliations:** 1 Ankara Kahramankazan State Hospital, Department of Pediatrics – Ankara, Turkey.; 2 Şanlıurfa Training and Research Hospital, Department of Pediatrics – Şanlıurfa, Turkey.; 3 Uşak University, Department of Pediatrics – Uşak, Turkey.; 4 University of Health Sciences, Mersin City Education and Research Hospital, Department of Child Health and Diseases – Mersin, Turkey.; 5 Istanbul University, Istanbul Faculty of Medicine, Department of Pediatrics, Division of Pediatric Nephrology – Istanbul, Turkey.

**Keywords:** Ambulance, Infant, Newborn, Emergency service, hospital

## Abstract

**OBJECTIVE:**

This study aimed to assess the reasons for admission, demographic and clinical characteristics, diagnostic procedures, hospitalization rates, and repeat visits among infants aged 0–12 months transported to the pediatric emergency department by 112 Emergency Medical Services ambulances in 2023, with a focus on. The study further evaluated the urgency of visits and sought to identify potentially preventable hospital admissions, while also examining the clinical decision-making processes involved.

**METHODS:**

This retrospective investigation involved infants brought to the Pediatric Emergency Department of Batman Training and Research Hospital from January 1 to December 31, 2023. Hospital records were examined for demographic reasons for admission, laboratory and imaging evaluations, hospitalization or referral outcomes, and repeat visits. Patients were grouped as neonates (0–28 days) or post-neonatal infants (29 days–12 months). Statistical analyses were conducted using Statistical Package for the Social Sciences 26.

**RESULTS:**

Of 3,950 ambulance transports, 957 (24.2%) involved infants aged 0–12 months; 55.6% were male. The leading causes for admission were respiratory distress (38.8%), jaundice (21.8%), and fever (8.3%). Hospitalization was needed in 60.6% of cases, and 20.0% were admitted to neonatal intensive care. Approximately 37.2% were discharged after clinical examination and limited diagnostic workup. Repeat presentations were seen in 11.3% of patients. Most admissions occurred on weekends and during off-hours periods.

**CONCLUSION:**

Many ambulance transports of infants involved non-urgent conditions, yet hospitalization rates remained high, especially among neonates. Improving parental education, scheduled follow-up for high-risk infants, and primary care access could reduce unnecessary visits.

## INTRODUCTION

The infant period is a highly vulnerable stage of life, characterized by ongoing physiological, metabolic, and environmental adaptation, with relatively higher risks of morbidity and mortality. The neonatal period, as the most delicate phase of infancy, is particularly significant, since illnesses may present with subtle clinical findings yet progress rapidly. In recent years, influenced by national health, economic, and social factors, shorter postpartum hospital stays have contributed to an increase in pediatric emergency department visits among neonates and infants, either due to emerging medical issues or for routine post-discharge evaluation^1^.

Emergency conditions are those with sudden onset that may be life-threatening or carry a risk of permanent health deterioration, thus requiring prompt medical attention^2^. In infancy, however, symptoms are often mild or nonspecific, which can lead to visits for non-urgent problems. This contributes to overcrowding in pediatric emergency departments and makes efficient healthcare delivery more challenging.

In recent years, neonatal intensive care unit admission rates have been reported to be approximately 8–10%, which is thought to reflect improvements in antenatal care and more structured perinatal monitoring processes^3^. Despite these advances in healthcare delivery, mortality during the neonatal and early infant periods continues to be largely associated with acute and rapidly developing conditions that require timely recognition and immediate medical intervention. Early diagnosis, close clinical follow-up, and prompt initiation of appropriate treatment remain critical factors in improving outcomes and reducing both mortality and morbidity rates in this vulnerable population^4^.

Prehospital emergency medical services, particularly 112 Emergency Medical Services ambulances, have an important role in identifying high-risk infants early and ensuring their rapid transfer to suitable healthcare facilities. Careful assessment of potential risks before discharge, along with comprehensive parental guidance and well-structured follow-up planning, can help reduce avoidable emergency department visits and repeated hospital admissions^5^. In this context, examining the reasons for admission, clinical features, and outcomes of infants aged 0–12 months transported to the pediatric emergency department by ambulance is essential for both improving neonatal health outcomes and supporting effective healthcare service planning.

## METHODS

This study was carried out within the scope of a research project conducted at Batman Training and Research Hospital and was approved by the Mardin Training and Research Hospital Scientific Research Ethics Committee (approval date: October 8, 2024; approval number: 2024/10-27). All study procedures were conducted in accordance with the principles outlined in the Declaration of Helsinki.

The study was planned as a retrospective, descriptive investigation. Data were obtained from the Hospital Information Management System of Batman Training and Research Hospital, and records of infants aged 0–12 months who were brought to the Pediatric Emergency Department by 112 Emergency Medical Services ambulances between January 1, 2023, and December 31, 2023, were reviewed in detail retrospectively.

Patients’ demographic features, reasons for admission, laboratory tests and imaging studies performed, hospitalization or referral status, and repeat emergency department visits were evaluated using data from the Hospital Information Management System. Visits by the same patient at different times were recorded as separate entries, and repeat admissions were assessed as a distinct variable during analysis.

During the study period, a total of 3,950 patients transported to the pediatric emergency department by 112 Emergency Medical Services ambulances were identified through the Hospital Information Management System. Among them, 957 infants aged 0–12 months formed the study group. No sampling method was applied, and all eligible cases were included directly in the analysis.

### Statistical analysis

Statistical analyses were performed using Statistical Package for the Social Sciences version 26. Continuous variables were expressed as mean±standard deviation or median (minimum–maximum), depending on their distribution. Categorical variables were presented as numbers (n) and percentages (%). Descriptive statistics were used to evaluate the demographic and clinical characteristics of the study population.

## RESULTS

In 2023, a total of 3,950 pediatric patients were transported to the pediatric emergency department by 112 Emergency Medical Services ambulances, and 957 of them were infants younger than one year (24.2%). The fact that infants under one year accounted for nearly one quarter of ambulance admissions may be related to the increased clinical vulnerability of this age group and their greater need for urgent medical evaluation. Among the included infants, 532 (55.6%) were male, and 425 (44.4%) were female (Table 1). The mean birth weight was 2,957.4 g, while the mean maternal age was 28.4 years.

**Table 1:** Distribution of gender and admission reasons among infants aged 0–12 months transported to the pediatric emergency department by 112 emergency medical services ambulances.

Variable	Groups	n	%
Gender	Male	532	55.6
	Female	425	44.4
	Total	957	100.0
**Reason for admission**		**n**	**%**
Respiratory distress		371	38.8
Jaundice		209	21.8
Fever		79	8.3
Feeding difficulties		67	7.0
Vomiting		56	5.9
Trauma		34	3.6
Constipation		26	2.7
Dermatological conditions		22	2.3
Diarrhea		14	1.5
Irritability and agitation		19	2.0
Cough		16	1.7
Surgical conditions		12	1.3
Cystitis		11	1.1
Neurological conditions		3	0.3
Other		18	1.9
Total		957	100.0

When the reasons for admission to the pediatric emergency department via 112 Emergency Medical Services ambulances were evaluated, respiratory distress was the most frequent complaint, accounting for 371 cases (38.8%). Jaundice followed as the second most common reason with 209 cases (21.8%), while fever ranked third with 79 cases (8.3%). Other presenting complaints included feeding difficulties, vomiting, trauma, constipation, dermatological problems, diarrhea, irritability and restlessness, cough, surgical conditions, cystitis, and neurological disorders (Table 1). The “other” category comprised conditions such as thyroid function abnormalities (elevated thyroid-stimulating hormone levels), sleep-related problems, conjunctivitis, prematurity, burns, and hypothermia.

Of the patients transported by 112 Emergency Medical Services ambulances, 580 (60.6%) were hospitalized. Among them, 116 (20.0%) required admission to the Neonatal Intensive Care Unit, most commonly due to respiratory distress, jaundice, and fever. A total of 356 patients (37.2%) who did not require hospitalization were discharged following clinical assessment and appropriate management. Additionally, 21 patients (2.2%) were referred to other centers for further diagnostic evaluation and treatment. The main reasons for referral were trauma or fall history (n=12), fever (n=5), severe respiratory distress (n=3), and jaundice (n=1).

When repeat visits to the pediatric emergency department were analyzed, 108 patients (11.3%) had at least one readmission during the study year via 112 Emergency Medical Services ambulances. Respiratory distress was the leading cause of these repeat admissions (59.3%), followed by jaundice (25.9%) and fever (14.8%).

The diagnostic tests and imaging methods used for patients evaluated in the pediatric emergency department are presented in Table 2. Laboratory blood tests were the most commonly requested investigations in this cohort. No diagnostic testing was performed in 105 patients (11.1%), while 566 patients (59.1%) underwent laboratory blood analysis. Among other diagnostic and imaging modalities, ultrasonography was used in 96 patients (10.0%), plain radiography in 67 patients (7.0%), urine analysis in 54 patients (5.6%), computed tomography in 43 patients (4.5%), and magnetic resonance imaging in 26 patients (2.7%).

**Table 2:** Diagnostic tests and imaging studies requested for infants aged 0–12 months transported to the pediatric emergency department by 112 emergency medical services ambulances.

Diagnostic test information	n	%
Laboratory blood tests requested	566	59.1
No diagnostic tests requested	105	11.1
Ultrasonography (USG) requested	96	10.0
Plain radiography requested	67	7.0
Urine tests requested	54	5.6
Computed tomography (CT) requested	43	4.5
Magnetic resonance imaging (MRI) requested	26	2.7
Total	957	100.0

When the timing of emergency department presentations among infants aged 0–12 months was examined, 283 admissions (29.6%) occurred on weekdays, while 674 (70.4%) took place on weekends and official holidays (Table 3). In terms of time of day, 108 presentations (11.3%) were recorded between 08:00 and 16:00, 617 (64.5%) between 16:00 and 24:00, and 232 (24.2%) between 24:00 and 08:00 (Table 3).

**Table 3:** Timing and distribution of admissions of infants aged 0–12 months transported to the pediatric emergency department by 112 emergency medical services ambulances.

Time of admission	n	%
Weekday admissions	283	29.6
Weekend and public holiday admissions	674	70.4
Total	957	100.0
**Time of admission**	**n**	**%**
Admissions between 08:00 and 16:00	108	11.3
Admissions between 16:00 and 24:00	617	64.5
Admissions between 24:00 and 08:00	232	24.2
Total	957	100.0

## DISCUSSION

In our study, analysis of the reasons for admission to the pediatric emergency department via 112 Emergency Medical Services ambulances showed that respiratory distress was the most frequent presenting complaint, with jaundice ranking second. A study conducted at the Çukurova University Faculty of Medicine Hospital similarly identified respiratory distress as the leading cause of emergency visits among preterm infants, which aligns with our findings^6^. In addition, Bozlu et al. reported that the most common reasons for neonatal admissions to the pediatric emergency department were jaundice, prematurity, and asphyxia, and that 78.4% of these patients required hospitalization^7^.

According to our findings, the mean birth weight of infants presenting to the pediatric emergency department was 2,957.4 g. In a similar study, the mean birth weight was reported as 3,232.22±456 g^8^. Among the infants included in our study, 532 (55.6%) were male and 425 (44.4%) were female, indicating a higher proportion of male patients. This finding is in line with previous studies evaluating pediatric emergency department admissions, which have also reported a higher frequency of male patients compared to females^9^.

In our study, the most common indications for admission to the neonatal intensive care unit were respiratory distress and jaundice. In contrast, another study in the literature reported that infants presenting to the emergency department with complaints such as fever and dehydration had higher hospitalization rates compared with other conditions. Based on these findings, it has been suggested that infants presenting with these clinical features should be admitted promptly for observation and treatment, without unnecessary delay in the emergency department for further diagnostic evaluation, provided their general condition is stable^10^.

As observed in this study, admissions of patients transported to the pediatric emergency department by 112 Emergency Medical Services ambulances occurred more frequently on weekends and public holidays than on weekdays. This pattern is consistent with previous literature describing the temporal distribution of pediatric emergency department visits. In a large-scale observational study by Schinkelshoek et al., a considerable proportion of pediatric emergency presentations were reported during out-of-office hours and on weekends, with higher patient volumes during these time periods^11^. Limited access to primary healthcare services outside regular working hours, increased parental concern when symptoms arise, and the inherent vulnerability of the neonatal period may all contribute to this pattern. In particular, symptoms such as respiratory distress, fever, and feeding difficulties in neonates often lead to heightened parental anxiety, which in turn increases the use of emergency medical services.

When the findings of this study are considered together with the existing literature, a considerable proportion of pediatric emergency department admissions via 112 Emergency Medical Services ambulances appears to occur during the early neonatal period. Previous studies have shown that emergency department visits among neonates are most commonly clustered within the first week of life^12^. This pattern reflects the physiological vulnerability of this early stage and the increased need for urgent medical care. The results emphasize the importance of providing comprehensive postnatal parental education and maintaining close follow-up for high-risk neonates during the early postnatal period.

## CONCLUSION

Infants represent a considerable proportion of pediatric emergency department admissions due to being in a highly vulnerable stage of life. In this study, a notable proportion of patients (37.2%) transported to the pediatric emergency department by 112 Emergency Medical Services ambulances were discharged following physical examination and basic clinical evaluation alone. This finding indicates that many emergency department visits may not correspond to true emergency conditions. In most cases, clinical assessment and routine laboratory investigations were sufficient for diagnosis and management, whereas the need for advanced imaging techniques and invasive procedures was limited.

The findings of this study suggest that insufficient parental education during the early postnatal period regarding physiological changes, routine well-child follow-up, and warning signs may be an important factor contributing to increased emergency department visits. In addition, early discharge practices and limited outpatient follow-up may also contribute to the rising number of pediatric emergency department admissions among infants aged 0–12 months. Strengthening postnatal parental education, establishing structured and regular outpatient follow-up for high-risk infants, and encouraging more appropriate use of primary healthcare services may help reduce unnecessary emergency department visits and repeat admissions.

## Data Availability

The datasets generated and/or analyzed during the current study are available from the corresponding author upon reasonable request.

## References

[1] Paul DA, Agiro A, Hoffman M, Denemark C, Brazen A, Pollack M (2016). Hospital admission and emergency department utilization in an infant medicaid population. Hosp Pediatr.

[2] Morgans A, Burgess SJ (2011). What is a health emergency? The difference in definition and understanding between patients and health professionals. Aust Health Rev.

[3] Sammour I, Karnati S, Othman H, Heis F, Peluso A, Aly H (2024). Trends in procedures in the neonatal intensive care unit. Am J Perinatol.

[4] Wang XL, Wang J, Yuan L, Shi WJ, Cao Y, Chen C (2018). Trend and causes of neonatal mortality in a level III children’s hospital in Shanghai: a 15-year retrospective study. World J Pediatr.

[5] Alpern ER, Stanley RM, Gorelick MH, Donaldson A, Knight S, Teach SJ (2006). Epidemiology of a pediatric emergency medicine research network: the PECARN Core Data Project. Pediatr Emerg Care.

[6] Atici A, Pirti M, Turkmen M, Narli N, Satar M (1996). Problems of neonates admitted to the emergency department. Turkiye Klinikleri J Pediatr.

[7] Bozlu G, Sagcan MF, Celik Y, Kuyucu N (2018). Retrospective evaluation of 624 neonates admitted to the pediatric emergency department. Mersin Univ Saglik Bilim Derg.

[8] Simsek Pervane S, Cicek C, Halil H (2024). Evaluation of neonatal profile presenting to the pediatric emergency department. Anatolian J Emerg Med.

[9] Rhinehart B, Sheridan D, Chung S, McGaughey S (2025). Neonatal presentations in the pediatric emergency department: a decade-long retrospective analysis. Am J Emerg Med.

[10] Gunes S, Sahin S, Koyuncu Arslan M, Karaca Dag O, Anil M (2022). Emergency room admission for newborns: how many are really urgent?. Forbes J Med.

[11] Schinkelshoek G, Borensztajn DM, Zachariasse JM, Maconochie IK, Alves CF, Freitas P (2020). Management of children visiting the emergency department during out-of-office hours: an observational study. BMJ Paediatr Open.

[12] Millar KR, Gloor JE, Wellington N, Joubert GI (2000). Early neonatal presentations to the pediatric emergency department. Pediatr Emerg Care.

